# *SlRCM1*, which encodes tomato Lutescent1, is required for chlorophyll synthesis and chloroplast development in fruits

**DOI:** 10.1038/s41438-021-00563-6

**Published:** 2021-06-01

**Authors:** Genzhong Liu, Huiyang Yu, Lei Yuan, Changxing Li, Jie Ye, Weifang Chen, Ying Wang, Pingfei Ge, Junhong Zhang, Zhibiao Ye, Yuyang Zhang

**Affiliations:** Key Laboratory of Horticultural Plant Biology, Ministry of Education, Huazhong Agricultural University, Wuhan, China

**Keywords:** Gene amplification, Agricultural genetics, Plant physiology, Plant breeding

## Abstract

In plants, chloroplasts are the sites at which photosynthesis occurs, and an increased abundance of chloroplasts increases the nutritional quality of plants and the resultant color of fruits. However, the molecular mechanisms underlying chlorophyll synthesis and chloroplast development in tomato fruits remain unknown. In this study, we isolated a chlorophyll-deficient mutant, *reduced chlorophyll mutant 1* (*rcm1*), by ethylmethanesulfonate mutagenesis; this mutant produced yellowish fruits with altered chloroplast development. MutMap revealed that *Solyc08g005010* is the causal gene underlying the *rcm1* mutant phenotype. A single-nucleotide base substitution in the second exon of *SlRCM1* results in premature termination of its translated protein. *SlRCM1* encodes a chloroplast-targeted metalloendopeptidase that is orthologous to the BCM1 protein of *Arabidopsis* and the stay-green G protein of soybean (*Glycine max* L. Merr.). Notably, the yellowish phenotype of the *lutescent1* mutant can be restored with the allele of *SlRCM1* from wild-type tomato. In contrast, knockout of *SlRCM1* by the CRISPR/Cas9 system in Alisa Craig yielded yellowish fruits at the mature green stage, as was the case for *lutescent1*. Amino acid sequence alignment and functional complementation assays showed that *SlRCM1* is indeed *Lutescent1*. These findings provide new insights into the regulation of chloroplast development in tomato fruits.

## Introduction

Fruit development is a complex and highly coordinated process that involves a series of specific physiological and biochemical changes^1,2^. During tomato fruit development, chloroplasts serve as sites of photosynthesis and carbohydrate accumulation and can be transformed into chromoplasts for carotenoid formation^3^. Chloroplast development in tomato plants is directly proportional to fruit development and nutrient accumulation. Both the synthesis and degradation of chloroplasts in plants are in a stable state of dynamic equilibrium. Therefore, it is of enormous importance to study the molecular mechanism underlying chloroplast development in the process of fruit ripening and development.

Chloroplast development has been demonstrated to be regulated by multiple transcription factors, among which *GLK2* is an influential transcription factor that regulates this process. Tomato fruits with full-length transcripts of *GLK2* mRNA exhibit a dark green shoulder that can promote photosynthesis and accumulate more nutrients, while *glk2* mutation eliminates this green shoulder^4^. *GLK2* overexpression resulted in dramatic upregulated expression of the *Solyc08g005010* gene, indicating a regulatory network involving chloroplast development^5^. The *TKN2* and *TKN4* genes in tomato modulate the gradient of chloroplast development in fruits by regulating *GLK2* expression^6^. Overexpression of the *APRR2-like* gene in tomato increased the content of chlorophyll in fruits^7^. *SlBBX20* in tomato regulates chloroplast development by modulating the expression of the *SlCAB1B*,*SlCAB6A* and *SlCHL27* genes^8^. *SlBEL11* encodes a transcription factor that negatively regulates chloroplast development and chlorophyll synthesis in tomato fruits. The SlBEL11 protein can directly bind to the promoters of genes involved in chloroplast development and chlorophyll synthesis,such as *TKN2*,*CAB* and *POR*, and downregulate the expression of these types of genes^9^. In plant growth and development, auxin signaling affects chlorophyll synthesis and chloroplast development. The auxin response factor ARF10 directly binds to the promoter of *GLK1* and activates its expression, thereby promoting chloroplast development and sugar accumulation^10^. SlARF6A directly binds to the promoters of the *GLK1*, *CAB1*, *CAB2* and *RbcS* genes and promotes their expression to modulate tomato fruit chloroplast development^11^. ARF2A, an auxin signal component, participates in fruit ripening and chloroplast development. In *Arabidopsis*, the *ARF2* gene is involved in the degradation of chlorophyll in leaves^12^. The chlorophyll content of *ARF2A-OX* transgenic fruit was significantly lower than that of wild-type fruit at 42 days post-anthesis^13^.

Chlorophyll synthesis in plants is a complex process involving 15 enzymes encoded by 27 genes^14^. Mg-chelatase catalyzes the binding of Mg^2+^ to protoporphyrin IX, which represents the first step in chlorophyll synthesis^15^, and Mg-protoporphyrin IX is further methylated by Mg-protoporphyrin IX methyltransferase, followed by four subsequent catalytic reactions to produce chlorophyll^16^. Chlorophyllide a oxygenase (CAO) catalyzes the conversion of *chlorophyll a* to *chlorophyll b* and plays an important role in the balance of *chlorophyll a* and *chlorophyll b*. Overexpression of *CAO* in tobacco promotes the synthesis of *chlorophyll b* and decreases the ratio of chlorophyll a and chlorophyll b^17^. GUN4, a porphyrin-binding protein, enhances the activity of Mg-chelatase by binding to protoporphyrin, which is the substrate of the Mg-chelatase reaction^18^. Overexpression of *GUN4* can significantly increase the chlorophyll content in tobacco leaves^19^. *SGR1*, encoding Mg dechelatase, promotes chloroplast degradation during plant maturation^20^. Mutation of *SGR1* in Chinese cabbage leads to increased chlorophyll concentrations and a stay-green phenotype^21^. In *Arabidopsis*, the *bcm1* mutant exhibits a pale-green leaf phenotype due to reduced chlorophyll contents. BCM1 interacts with GUN4 to enhance Mg-chelatase activity. BCM1 can also interact with SGR1 to destabilize the SGR1 protein^22^. GluTR is a glutamyl tRNA reductase involved in porphyrin and chlorophyll biosynthesis^23^. Interactions between BCM1 and GluTR affect the synthesis of 5-aminolevulinic acid, a key precursor in the biosynthesis of porphyrin during chlorophyll synthesis^22^. The product of *CHLM* is magnesium protoporphyrin IX methyltransferase that converts Mg-protoporphyrin IX to Mg-protoporphyrin IX methylester during chlorophyll synthesis^24^. BCM1 interacts with CHLM to promote the formation of a MgCH-GUN4-CHLM enzyme complex^22^. The stay-green *G* gene is a homologous gene of *BCM1* in soybean; this gene positively regulates chlorophyll synthesis in the soybean seed coat^25^. Therefore, *BCM1* plays an important and conserved role in chlorophyll synthesis in different crop species.

In tomato, the *Lutescent2* (*L2*) gene, which encodes a zinc metalloprotease, has been characterized, and this gene has been shown to regulate chloroplast development and fruit maturity^26^. Similarly, the number of chloroplasts per cell in immature green fruit pericarp tissue of *lutescent1* (*l1*) mutant was reduced significantly compared with that of the wild type. In addition, fully expanded leaflets of the *l1* mutant exhibited a more dramatic yellowish phenotype^26^. It was previously reported that the chlorophyll content of the fruits of the *l1* mutant was reduced, leading to a senescent phenotype for *l1*^27^. Chloroplast development is hindered in *l1*^28^, although the causative gene underlying chloroplast defects and chlorophyll reduction in *l1* has not yet been identified.

In this study, we obtained a reduced chlorophyll mutant (*rcm1*) of tomato; this mutant is an *l1* allelic mutant with altered chloroplast development and was generated via ethylmethanesulfonate (EMS) mutagenesis. We discovered via BSA + DNA-Seq and MutMap that the *rcm1* mutant carries a single-nucleotide polymorphism (T → A), resulting in premature termination of the SlRCM1 protein. Sequence analysis and functional characterization showed that the *SlRCM1* gene, located at the *Lutescent1* locus, encodes an ortholog of the BCM1 protein of *Arabidopsis* and the stay-green G protein of soybean (*Glycine max* L. Merr.). *SlRCM1* regulates chlorophyll synthesis and chloroplast development in fruits at the mature green and red ripe stages, while its ortholog regulates chlorophyll synthesis in the leaves of *Arabidopsis* and seeds of soybean^22,25^. These findings highlight the molecular mechanisms underlying chlorophyll synthesis and chloroplast development in tomato fruits.

## Results

### The tomato *rcm1* mutant exhibits altered chloroplast development

**Fig. 1:**
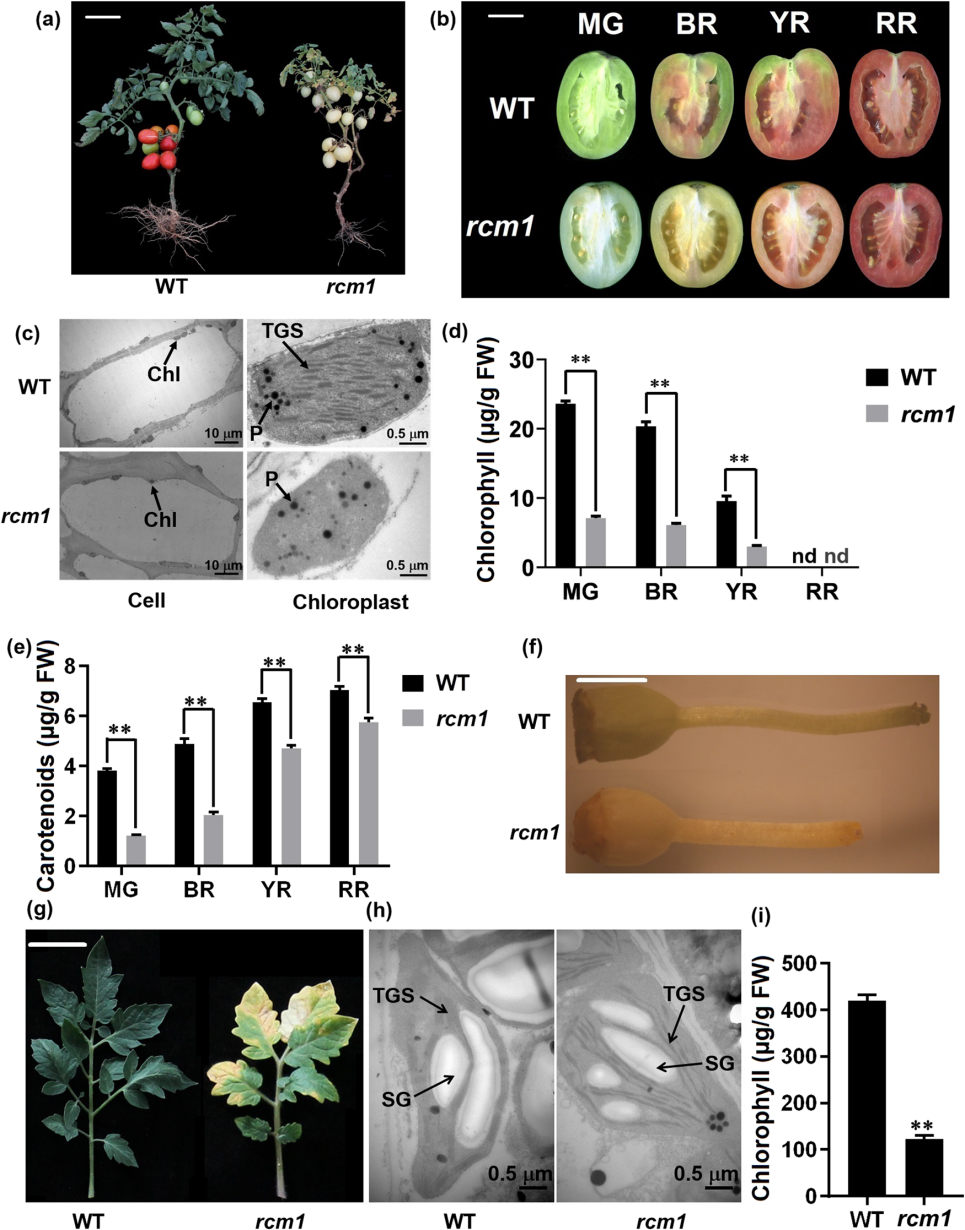
Phenotypes of wild-type and *rcm1* mutant plants. **a** Whole-plant morphology of the reduced-chlorophyll mutant (*rcm1*) and the wild type (Ligeer 87-5). Scale bars, 15 cm. **b** Phenotypes of Wild Type (WT) and *rcm1* tomato fruits at four developmental stages. MG, mature green stage; BR, breaker stage; YR, yellow ripe stage; RR, red ripe stage. Scale bars, 2 cm. **c** Chloroplast ultrastructure of mature green fruits of wild-type and *rcm1* mutant plants via transmission electron microscopy. P, plastoglobuli; TGS, thylakoid granum stacks. Scale bars, 10 μm (left) and 0.5 μm (right). **d** Chlorophyll content of fruit pericarps from wild type and *rcm1* at the MG, BR, YR and RR stages. The data are presented as the means ± SDs (*n* = 6). **e** Carotenoid contents of fruit pericarps from wild type and *rcm1* at the MG, BR, YR and RR stages. The data are presented as the means ± SDs (*n* = 6). **f** Phenotypes of the pistils of the wild type and *rcm1* mutant. Scale bars, 2 mm. **g** Morphology of the leaves of the *rcm1* mutant and the wild type. Scale bars, 2 cm. **h** Chloroplast ultrastructure of leaves of wild-type and *rcm1* plants using transmission electron microscopy. SG, starch grains; TGS, thylakoid granum stacks. Scale bars, 0.5 μm. **i** Chlorophyll content of leaves from wild-type and *rcm1* plants. The data are presented as the means ± SDs (*n* = 6). The asterisks indicate statistically significant differences according to *t*-tests: **, *P*-value < 0.01. nd, not detected

Seeds of the tomato cultivar Ligeer 87-5 were treated with 1% EMS to obtain novel mutants. A reduced chlorophyll mutant (*rcm1*) was isolated from the EMS-mutagenized population (Fig. 1a). The fruits of the *rcm1* mutant exhibited reduced amounts of chlorophyll relative to those of the wild type. The fruits at the mature green (MG) and breaker (BR) stages of the *rcm1* mutant showed a sharp decrease in chlorophyll relative to those of the wild type. However, the fruits of the *rcm1* mutant could still turn red at the red ripe (RR) stage (Fig. 1b). Furthermore, the thylakoid membranes were collapsed in the chloroplasts of fruits of the *rcm1* mutant at the mature green stage (Fig. 1c). The chloroplasts in the *rcm1* fruits were smaller than those of the wild type (Fig. 1c). Subsequently, the chlorophyll content in the *rcm1* mutant fruits was significantly lower than that in the wild-type fruits at the MG, BR and yellow red (YR) stages, and no chlorophyll was detected at the RR stage (Fig. 1d). The carotenoid content in the *rcm1* mutant fruits was also significantly lower than that in the wild-type fruits at all four developmental stages (Fig. 1e). The a*/b* colorimetric values (Fig. S1a), which are proportional to the lycopene, and total soluble solids contents (Fig. S1b) of red ripe fruits of the *rcm1* mutant decreased relative to those of the wild type. Fruit ripening of the *rcm1* mutant was delayed by approximately 6 days compared to that of the wild-type (Fig. S2a), and the ethylene production rate of the *rcm1* mutant fruits was lower than that of the wild-type fruits (Fig. S2b).

To fully evaluate the phenotype of the *rcm1* mutant, the leaves and pistils were compared between the *rcm1* mutant and wild type. A lack of chlorophyll accumulation was observed in the developing pistils and leaves of *rcm1* relative to the wild type (Fig. 1f, g). Transmission electron microscopy revealed impaired development of thylakoid membranes in the chloroplasts of the *rcm1* mutant compared to those of the wild type (Fig. 1h). Accordingly, the chlorophyll content in the *rcm1* mutant leaves was significantly lower than that in the wild-type leaves (Fig. 1i). Compared to that of the wild type, the maximum photochemical efficiency (Fv/Fm) of leaves of the *rcm1* mutant decreased significantly (Fig. S3a, b). Additionally, the quantum efficiency of PSII (Y(II)) of the leaves of the *rcm1* mutant was significantly impaired compared with that of the wild type (Fig. S3c). Additionally, pollen vitality in *rcm1* was lower than that in Ligeer 87-5 (WT); the percentage of malformed pollen in *rcm1* was greater than that in the wild type (Fig. S4).

### Cloning of *SlRCM1*

To genetically characterize the yellowish fruit phenotype of the *rcm1* mutant, we generated an F_2_ population consisting of 307 individuals by crossing Ligeer 87-5 with the *rcm1* mutant. The F_1_ generation of the cross between Ligeer 87-5 and *rcm1* displayed a normal phenotype similar to that of Ligeer 87-5, which suggested that the gene underlying the reduced chlorophyll phenotype of the *rcm1* mutant is a recessive gene. In the F_2_ population, the ratio between the number of individuals with a normal phenotype (237) and the number of individuals with a chlorophyll-deficient phenotype (70) was approximately 3:1 (χ^2^ = 0.68, χ^2^_0.05_ = 3.84), indicating that the chlorophyll-deficient phenotype was controlled by a single gene (Table S1).

**Fig. 2:**
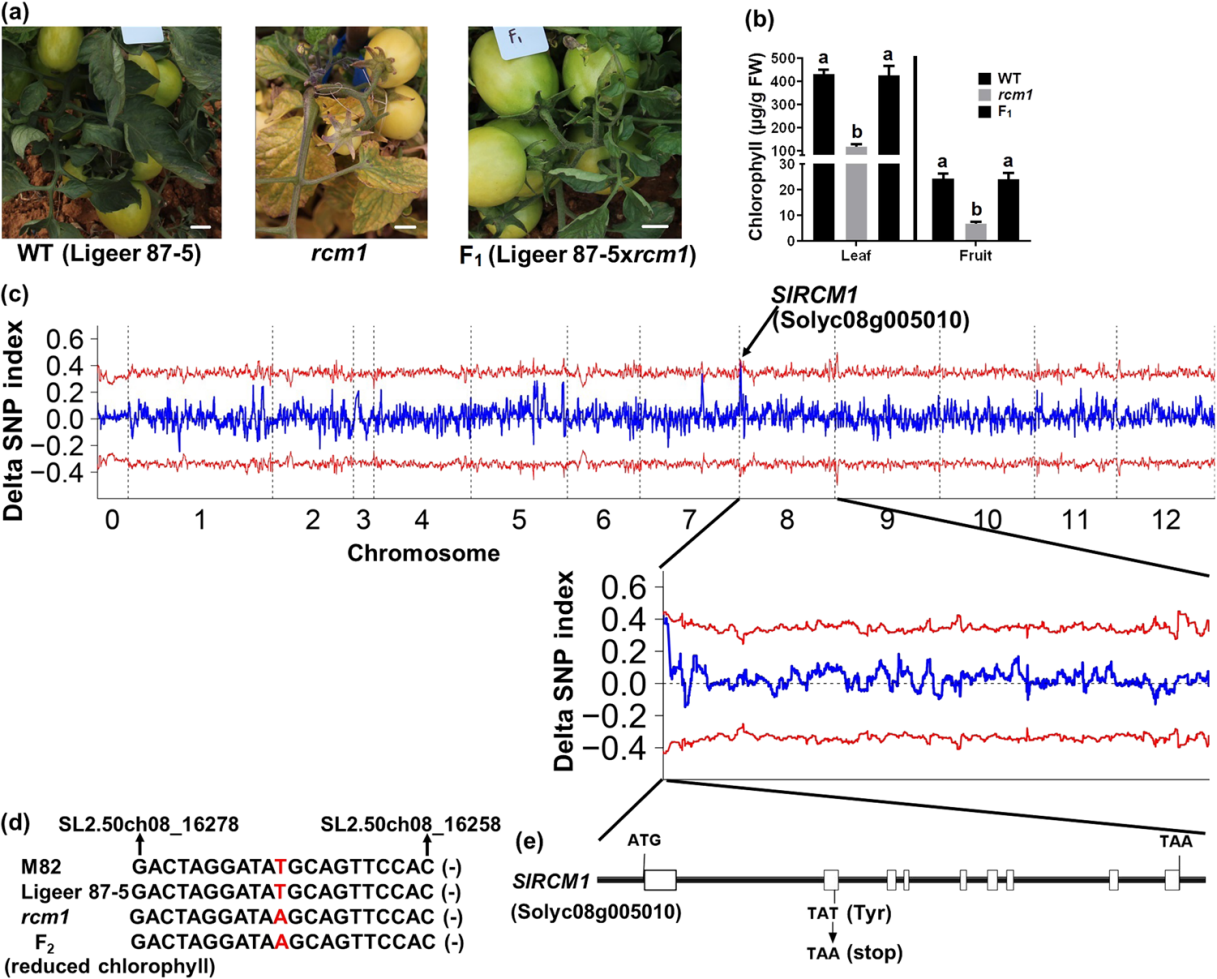
Gene cloning, sequence variation and structure of the *SlRCM1* gene. **a** Phenotypes of leaves and fruits from the wild-type and *rcm1* mutant plants and their F_1_ progeny. Scale bars, 2 cm. **b** Chlorophyll content of mature leaves and MG-stage fruits from the wild type and *rcm1* mutant and their F_1_ progeny. The data are presented as the means ± SDs (*n* = 3). The means followed by different letters indicate statistical significance at *P* = 0.05. **c** Differences in allele frequencies between 25 lines with green fruits and 25 lines with yellowish fruits in the F_2_ population. The X axis represents the 12 chromosomes of tomato. The Y axis represents the difference in allele frequencies (SNP values) between the two pools. The red line represents the 95% confidence interval. The SNPs associated with the 95% confidence interval are located between SL2.50ch08_1 and SL2.50ch08_1010000. The blue line represents the average value of the ΔSNP index per 1 Mb window. **d** The fragment containing SNP (SL2.50ch08_16268) was subjected to PCR amplification and Sanger sequencing. The red letters indicate the bases of M82 (T), Ligeer 87-5 (T), *rcm1* (A) and members of the hybrid F_2_ generation (A) with reduced chlorophyll at SL2.50ch08_16268. “-“ indicates the antisense strand of the genome. **e** Structure of the *SlRCM1* gene. The black lines represent introns and untranslated regions. The white boxes represent exons. The T (WT)-to-A (*rcm1*) substitution in the second exon of *SlRCM1* caused a conversion from Tyr (WT) to a stop codon (*rcm1*)

Next, we performed BSA and MutMap analyses to isolate the candidate genes. We sequenced two bulk populations comprising 25 individuals with green fruit or yellowish fruit at the mature green stage. Each library was sequenced at a depth of a ×25 genome equivalent. The generated reads were mapped to the tomato reference genome (M82), and allele frequency differences of 40,124 SNPs from the two pools were calculated and mapped across the 12 chromosomes in tomato to form a Manhattan plot (Fig. 2c). The confidence threshold exceeded 95% only at the beginning of chromosome 8, between SL2.50ch08_1 and SL2.50ch08_1010000 (Table S2). Generally, single-base mutations are frequently generated by EMS mutagenesis^29,30^. There were six SNPs between the two pools between SL2.50ch08_1 and SL2.50ch08_1010000 (Table S3). Genetic analysis of the segregating populations indicated that the phenotype of the *rcm1* mutant is likely controlled by a single locus. Since M82 (the genotype of the reference genome) develops normal chloroplasts in its fruits, SNPs in the recessive pool with an allele frequency of 1 and different from the reference genome (M82) were scored, and only one SNP (A → T) at SL2.50ch08_16268 out of all SNPs in the coding regions was identified.

Furthermore, no InDels or other structural variations were detected via 25 equivalent genome sequencing events of the WT and *rcm1* mutants. We analyzed the allele frequency of the SNP at SL2.50ch08_16268 in both pools with wild-type and mutant phenotypes. The SNP at SL2.50ch08_16268 in M82 was A, whereas in the mutant pool, it was T (100%) (Table 1). Furthermore, the SNP at SL2.50ch08_16268 in 30 F_2_ progeny with yellowish fruit was T (100%), which was confirmed via PCR and Sanger sequencing. This SNP (SL2.50ch08_16268) occurs in the second exon of *Solyc08g005010* and was further verified by Sanger sequencing. Because the transcriptional direction of *Solyc08g005010* is opposite to that of the genome, the nucleotide of SL2.50ch08_16268 in the coding strand of *Solyc08g005010* in M82 and Ligeer 87-5 was T, but it was A in the *rcm1* mutant (Fig. 2d, e). The substitution of T (wild type) to A (*rcm1* mutant) resulted in the conversion of Tyr (wild type) to a stop codon (*rcm1* mutant) (Fig. 2e), indicating that the translation of the SlRCM1 protein was terminated prematurely in the *rcm1* mutant. The predicted protein encoded by the *SlRCM1* allele from the *rcm1* mutant did not include any functional domain (Fig. S5a). Therefore, we inferred that the *SlRCM1* allele in the *rcm1* mutant is null. Furthermore, the expression level of the *SlRCM1* allele in the *rcm1* mutant was equivalent to that in the wild type (Fig. S5b). Therefore, we considered *Solyc08g005010* to be the candidate *SlRCM1* gene, which encodes a CAAX-type endopeptidase.

**Table 1:** List of candidate SNPs and genes between SL2.50ch08_1 and SL2.50ch08_1010000

Chr	Pos	Ref	Allele	Wild type index	Mutant index	Delta SNP index	Location	Gene	Direction	Amino acid change
SL2.50ch08	16268	A	T	0.27	1	0.73	Exon	Solyc08g005010	Reverse	Tyr→Stop
SL2.50ch08	394612	G	A	0.42	1	0.58	Intergene	--	--	--

### The yellowish phenotype of fruits of *rcm1* was complemented by *SlRCM1* from Ligeer 87-5

**Fig. 3:**
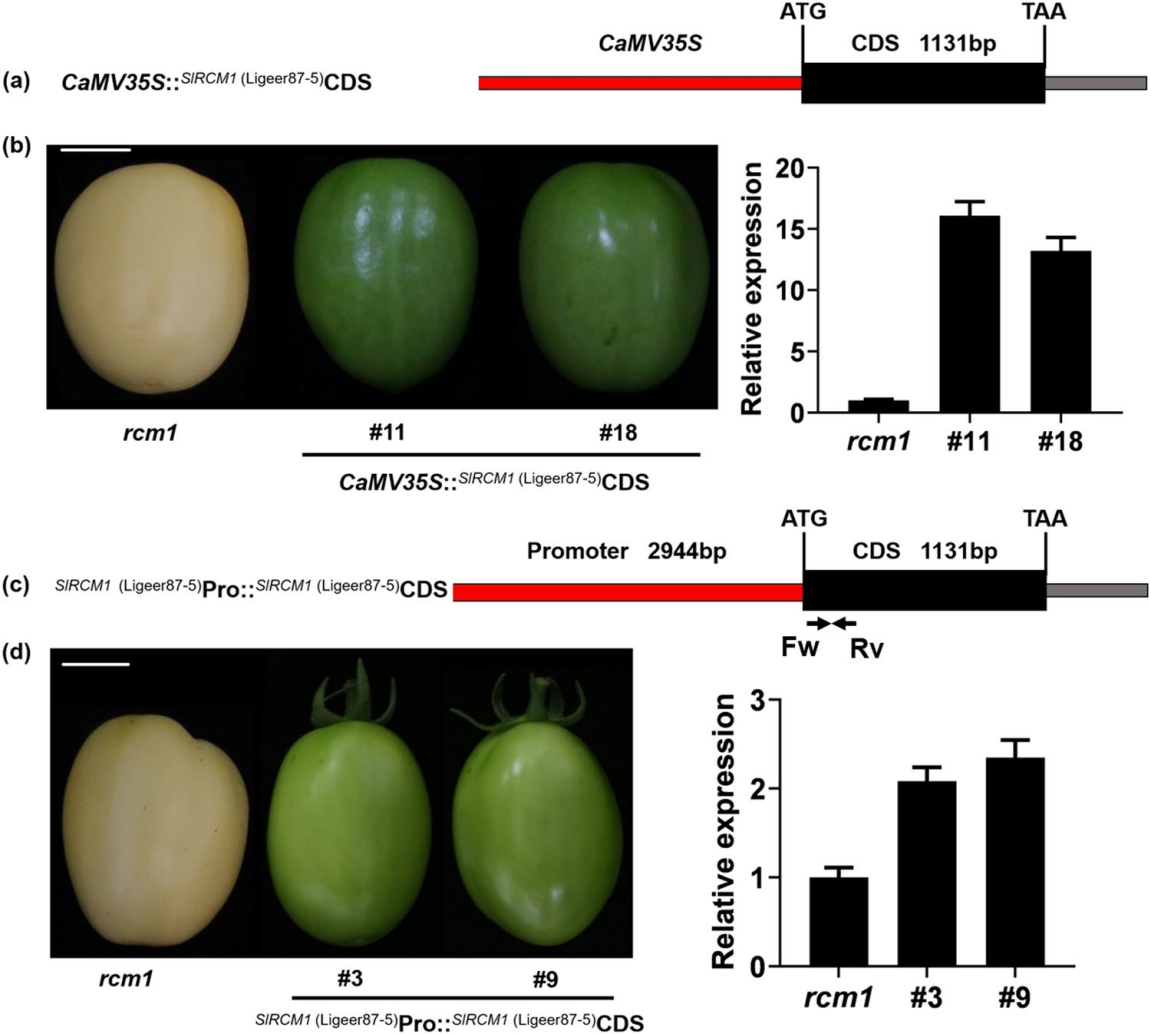
The *CaMV35S* promoter and native promoter drive *SlRCM1* to restore normal chlorophyll synthesis in *rcm1*. **a** Schematic structure of the expression vector with *SlRCM1* driven by the *CaMV35S* promoter. **b** Overexpression of *SlRCM1* driven by the *CaMV35S* promoter in *rcm1*. Overexpressing *SlRCM1* in *rcm1* rescued the phenotype, as was the case for Ligeer 87-5. The relative expression of *SlRCM1* in mature green fruits of *SlRCM1*-overexpressing lines and untransformed control (*rcm1*) plants was quantified. Scale bars, 2 cm. **c** Schematic structure of the expression vector with *SlRCM1* driven by its native 2944-bp promoter from Ligeer 87-5. The two arrows represent the primers used to analyze the transcript level of *SlRCM1*. Fw: Forward primer; Rv: Reverse primer. **d** The phenotype of the fruits of the transgenic lines could be restored to that of Ligeer 87-5. The relative expression of *SlRCM1* in mature green fruits was quantified in the transgenic lines and *rcm1*. Scale bars, 2 cm. The *ACTIN* gene (*Solyc11g005330*) was used as the internal control. The data are presented as the means ± SDs (*n* = 3)

To verify whether *SlRCM1* is the causal gene for the chlorophyll-deficient phenotype of the *rcm1* mutant, *SlRCM1* was overexpressed (OE) under the control of the *CaMV35S* promoter in the *rcm1* mutant (Fig. 3a). Nineteen independent transgenic lines were obtained. The expression levels of the *SlRCM1* gene in the OE-11 (#11) and OE-18 (#18) lines in the T_1_ generation significantly increased relative to those of the *rcm1* mutant. The normal-green phenotypes of the fruits of the OE-11 and OE-18 lines were restored at the mature green stage (Fig. 3b). To further verify the biological function of *SlRCM1*, a transformation construct (*Pro* ^*SlRCM1* (Ligeer 87-5)^::CDS^*SlRCM1* (Ligeer 87-5)^) was prepared by inserting the *SlRCM1* CDS from Ligeer 87-5 into a pHELLSGATE8 vector under the native promoter of the transgene (Fig. 3c). We introduced this construct into the *rcm1* mutant by *Agrobacterium*-mediated transformation. Eleven independent transgenic plants were identified. The phenotype of the *rcm1* transgenic lines was restored to that of the wild type (normal green fruit) (Fig. 3d). Overall, these results confirmed that *SlRCM1* is the correct candidate gene.

### *SlRCM1* regulates chloroplast development in tomato

**Fig. 4:**
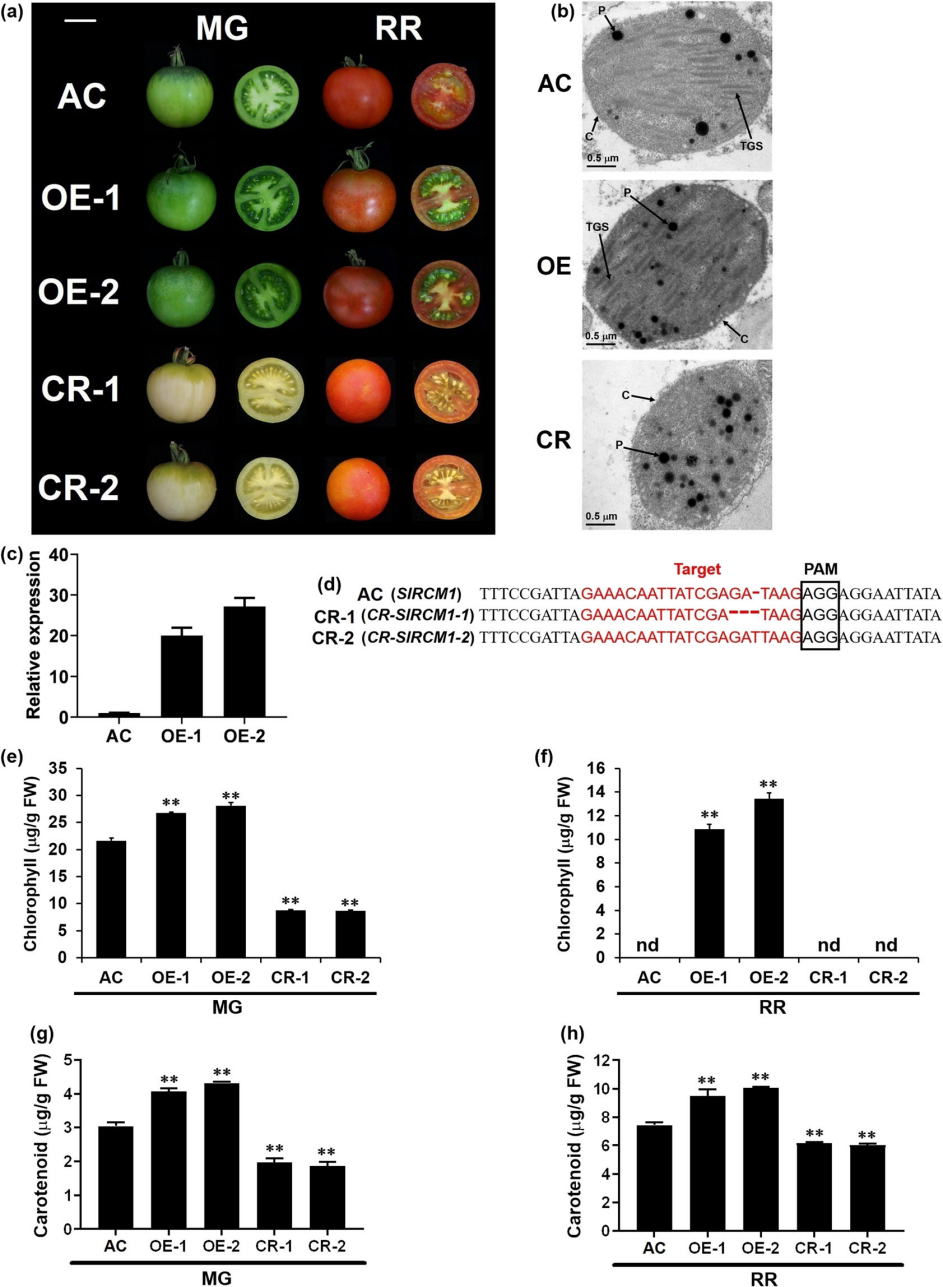
Phenotypes of *SlRCM1* transgenic lines and wild-type plants. **a** Fruits of AC, *SlRCM1* overexpression and knockout lines at the mature green (MG) and red ripe (RR) stages. Scale bars, 3 cm. **b** The structures of chloroplasts in mature green fruits of AC, *SlRCM1*-OE (OE) and *SlRCM1*-CRISPR (CR) were observed via transmission electron microscopy. C, chloroplast; P, plastoglobulus; TGS, thylakoid granum stack. Scale bars, 0.5 μm. **c** The relative expression of *SlRCM1* in mature green fruits of *SlRCM1* overexpression lines and the control (AC). The *ACTIN* gene (*Solyc11g005330*) was used as the internal control. The data are presented as the means ± SDs (*n* = 3). **d** Mutation types of *SlRCM1* knockout lines in the T_1_ generation were identified. **e** Chlorophyll content in the fruit pericarps of AC, *SlRCM1*-OE (OE) and *SlRCM1*-CRISPR (CR) lines at the mature green (MG) stage. **f** Chlorophyll content in the fruit pericarps of AC, *SlRCM1*-OE (OE) and *SlRCM1*-CRISPR (CR) lines at the red ripe (RR) stage. **g** Carotenoid content in the fruit pericarps of AC, *SlRCM1*-OE (OE) and *SlRCM1*-CRISPR (CR) lines at the MG stage. **h** Carotenoid content in the fruit pericarps of AC, *SlRCM1*-OE (OE) and *SlRCM1*-CRISPR (CR) lines at the RR stage. The data are presented as the means ± SDs (*n* = 6). The asterisks indicate statistically significant differences according to *t*-tests: **, *P*-value < 0.01. nd, not detected

To further verify that *SlRCM1* regulates chloroplast development in tomato fruits, we overexpressed the *SlRCM1* gene in *S. lycopersicum* (L.) cv. Alisa Craig (AC) by *Agrobacterium*-mediated transformation. Compared to those of the wild type, the fruits of the OE-1 and OE-2 lines appeared dark green at the mature green stage (Fig. 4a). The number of thylakoids and thylakoid granum stacks in mature green fruits of the OE lines was higher than that of the wild type (Fig. 4b). Interestingly, the locular material surrounding the seeds remained green, and chlorophyll could still be detected at the red ripe stage in the overexpression lines (Fig. 4a). Indeed, the expression levels of the *SlRCM1* gene in the OE-1 and OE-2 lines were significantly higher than those in the wild-type (AC) line (Fig. 4c). The chlorophyll content in the MG- and RR-stage fruits of OE-1 and OE-2 lines increased significantly relative to that in the wild type (Fig. 4e, f). In addition, the carotenoid content in the MG- and RR-stage fruits of the OE-1 and OE-2 lines increased significantly relative to that of the wild type (Fig. 4g, h). Chlorophyll in the RR-stage fruit of AC was not detected. Furthermore, we used CRISPR/Cas9 (CR) to edit the first exon of *SlRCM1* in the AC background (Fig. 4a, d). We determined the type of mutation in the T_1_ generation using PCR and Sanger sequencing. The *SlRCM1* knockout line CR-1 contained a 2-bp deletion in *SlRCM1*, and the knockout line CR-2 contained a 1-bp insertion in *SlRCM1* (Fig. 4d). Both the CR-1 and CR-2 lines exhibited a reduced chlorophyll phenotype at and after the mature green stage (Fig. 4a). Furthermore, the development of thylakoid membranes was impaired in the mature green fruits of the CR lines compared to that of the wild type (Fig. 4b). The chlorophyll content in the MG-stage fruits of CR-1 and CR-2 plants decreased significantly relative to that in the wild-type plants (Fig. 4e). These results indicate that *SlRCM1* is responsible for chlorophyll synthesis and chloroplast development in tomato fruits.

### SlRCM1 is Lutescent1

**Fig. 5:**
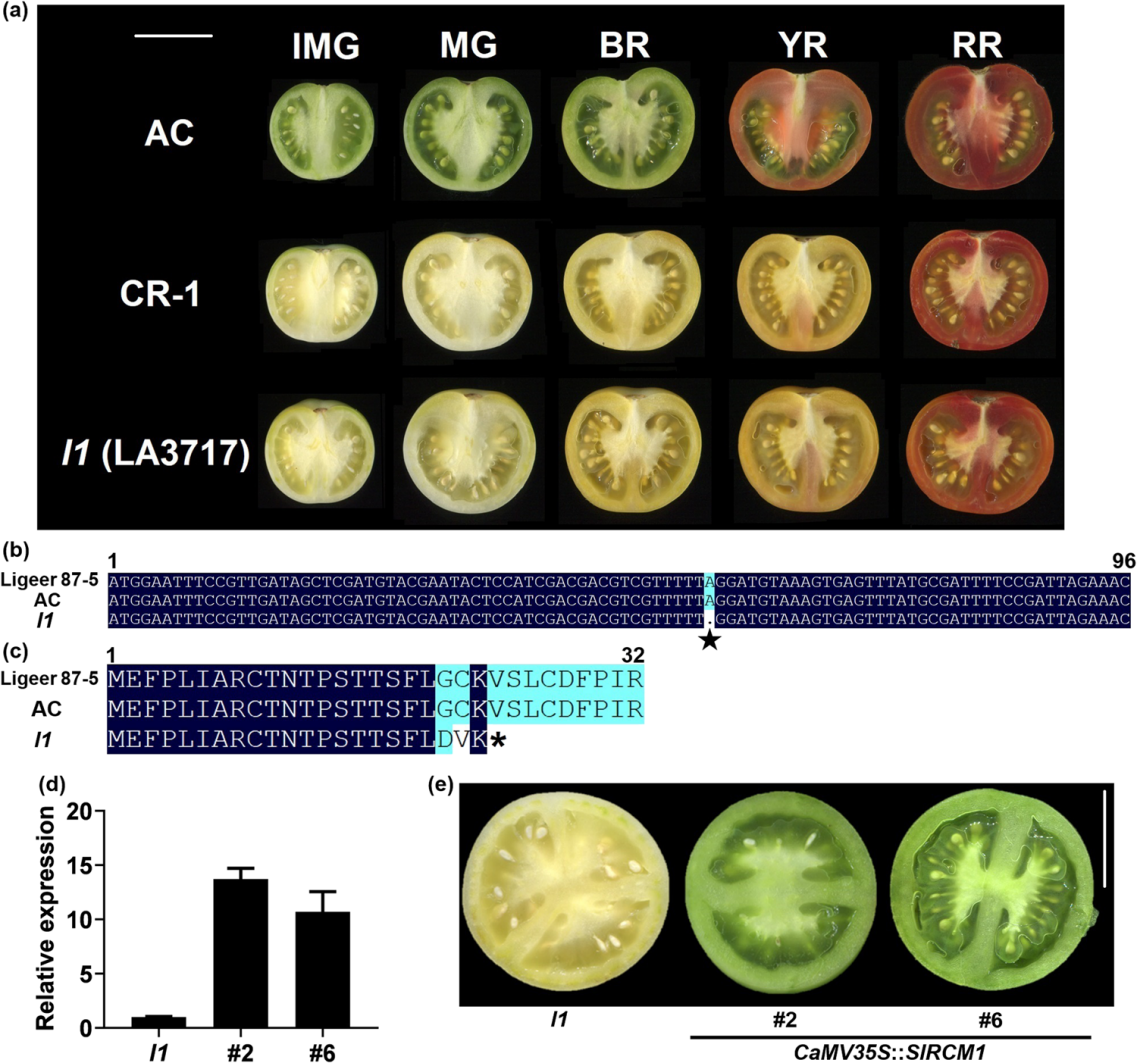
*Lutescent1* is the allele of *SlRCM1*. **a** Phenotypes of fruits from the CR-1 line, *lutescent1* mutant (*l1*) and AC at different developmental stages. IMG, immature green stage; MG, mature green stage; BR, breaker stage; YR, yellow ripe stage; RR, red ripe stage. The *l1* is a mutant in the AC background. Scale bars, 3 cm. **b** Nucleotide sequence alignment of *SlRCM1* fragments in Ligeer 87-5, Ailsa Craig (AC) and *l1*. The other bases are consistent among Ligeer 87-5, AC and *l1*. The black star indicates the base at SL2.50ch08_16268 in the first exon of the *SlRCM1* gene. **c** Amino acid sequence alignment of the SlRCM1 protein of Ligeer 87-5, AC and *l1*. **d** Relative expression of *SlRCM1* in mature green fruits of the *SlRCM1-*overexpressing lines and the control (*l1*). The *ACTIN* gene (*Solyc11g005330*) was used as an internal control. The data are presented as the means ± SDs (*n* = 3). **e** Complementation of the *l1* mutant using *SlRCM1*. Phenotypes of mature green fruits from *l1-* and *SlRCM1*-overexpressing lines were imaged and are shown. Scale bars, 3 cm

A spontaneous *lutescent1* mutant (*l1*), LA3717, was derived from Ailsa Craig (AC). The *l1* mutation has been shown to dramatically affect tomato fruit development. *l1* mutants have a low chlorophyll content in their fruits, especially under high-light and dark conditions, which enhances the rate of chlorophyll loss^26^. To date, the gene underlying the *l1* mutant has not yet been cloned. The fruits of *SlRCM1* knockout lines (CR) generated using the CRISPR/Cas9 system exhibited the same yellowish phenotype as did the fruits of the *l1* mutant in the Alisa Craig (AC) background described in the Tomato Genetics Resource Center (TGRC; https://tgrc.ucdavis.edu) (Fig. 5a). The yellowish phenotype of the fruits of the *l1* mutant is purportedly controlled by a single gene located at the beginning of chromosome 8^26,31^. We used *SlRCM1-*specific primers to amplify full-length gDNA and the 5-kb promoter of *SlRCM1* from AC, Ligeer 87-5 and *l1* (Table S4). A base deletion led to premature termination of the SlRCM1 protein in *l1* (Fig. 5b, c). Furthermore, the expression level of the *SlRCM1* allele in the *l1* mutant was equivalent to that in AC (Fig. S5c). SlRC*M1* was subsequently overexpressed under the control of the *CaMV35S* promoter in the *l1* mutant. Nine independent transgenic lines were identified. The expression level of the *SlRCM1* gene in the 2^nd^ (#2) and 6^th^ (#6) transgenic lines was significantly higher than that in the control (*l1*) (Fig. 5d). Functional complementation of *SlRCM1* in *l1* restored normal plant growth and development (Fig. 5e). Taken together, these results indicate that *SlRCM1* may be a causal gene underlying the yellowish phenotype of the *l1* mutant.

### *SlRCM1* expression and subcellular localization

**Fig. 6:**
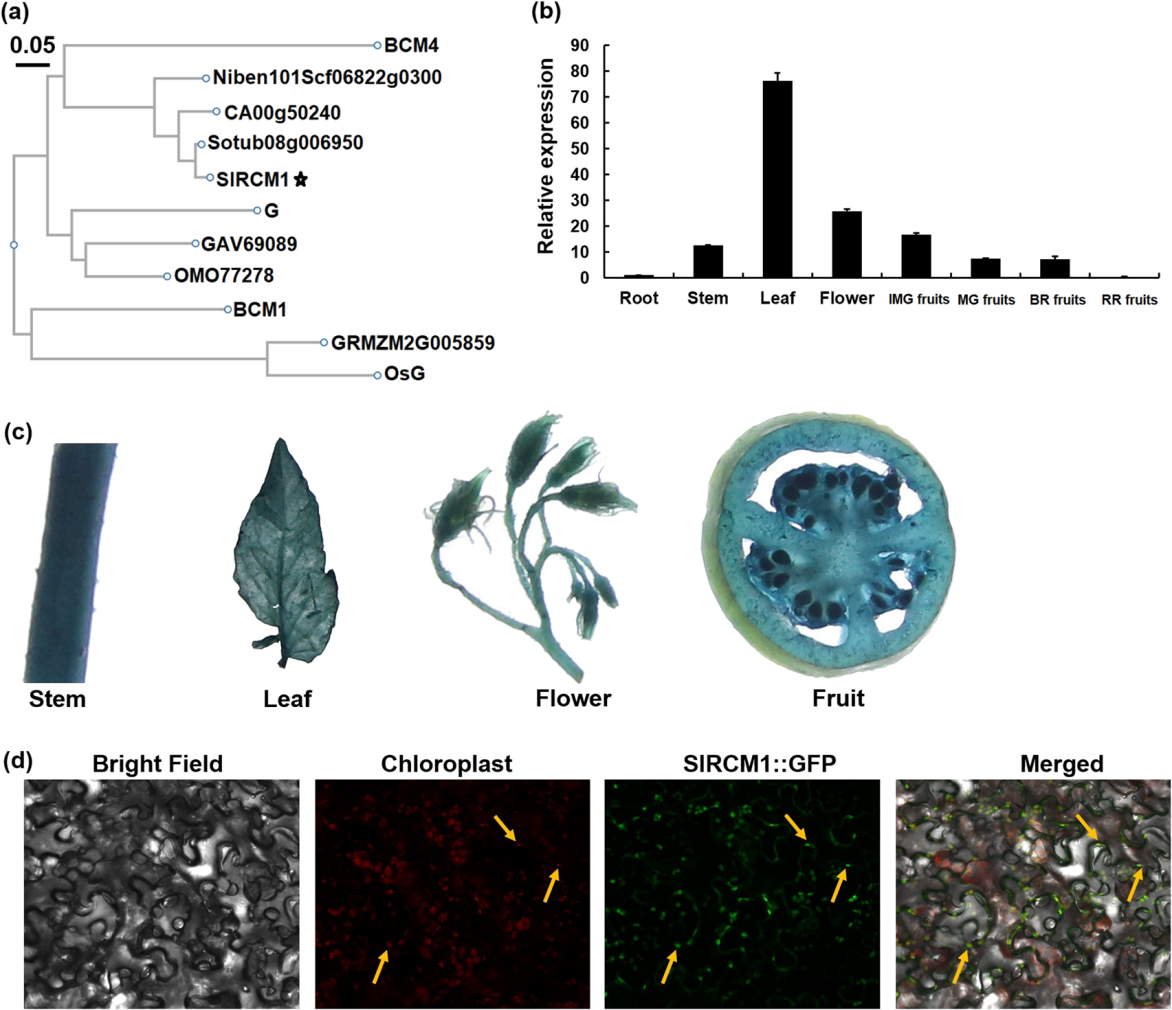
Characterization of *SlRCM1*. **a** Phylogenetic analysis of *SlRCM1*. The tomato SlRCM1 protein sequence was used to query (via BLAST) the homologous proteins of different species such as soybean (G), potato (Sotub08g006950), pepper (CA00g50240), tobacco (Niben101Scf06822g03002), *Arabidopsis thaliana* (BCM1 and BCM4), rice (OsG), maize (GRMZM2G005859), jute (OMO77278), and *Cephalotus follicularis* (GAV69089) (http://www.ncbi.nlm.nih.gov/). **b** Relative expression of *SlRCM1* in different tissues of Alisa Craig (AC) by qRT-PCR. RNA was extracted from the roots, stems, leaves, flowers, immature green fruits (IMG), mature green fruits (MG), breaker-stage fruits (BR) and red ripe fruits (RR). The *ACTIN* gene (*Solyc11g005330*) was used as the internal control. The data are presented as the means ± SDs (*n* = 3). **c** The *SlRCM1* promoter drives *GUS* gene expression in AC. Tissues of transgenic tomato plants were stained with X-Gluc. **d** Subcellular localization of the SlRCM1 protein. SlRCM1::GFP fusion proteins were transiently expressed in tobacco leaves. Chloroplasts exhibit red fluorescence under a confocal microscope. The arrows highlight the positions of the red and green fluorescence. Scale bars, 25 μm

The gDNA of the *SlRCM1* gene is 6,299 bp in length and consists of nine exons and eight introns. The *SlRCM1* gene encodes a protein comprising 376 amino acids. Alignment of amino acid sequences (Fig. S6) and phylogenetic analysis (Fig. 6a) showed that the SlRCM1 protein is highly conserved among soybean, potato, pepper, tobacco, *Arabidopsis*, rice, maize, jute and *Cephalotus follicularis*, suggesting that the SlRCM1 protein may play an essential role in plants. Tomato *SlRCM1* may function in chloroplast development and chlorophyll synthesis through a conserved biological process, similar to that in soybean and *Arabidopsis thaliana*.

To investigate the expression pattern of *SlRCM1*, quantitative reverse transcription polymerase chain reaction (qRT-PCR) was performed as described previously^32^. *SlRCM1* expression was detected in all tissues, and relatively high expression levels were detected in the leaves, flowers and immature green fruits (Fig. 6b). Furthermore, we used GUS staining to study the expression of *SlRCM1* in several tissues. The GUS staining revealed that *SlRCM1* was expressed in the stems, leaves, flowers and fruits (Fig. 6c).

To determine the SlRCM1 subcellular localization, we constructed a SlRCM1::GFP fusion protein. Through *Agrobacterium*-mediated infiltration, the SlRCM1::GFP fusion protein was transiently expressed in tobacco. Under a Nikon e600 fluorescence microscope (Nikon, Tokyo, Japan), the autofluorescence of the chloroplast appeared red^33^. The SlRCM1::GFP fluorescence overlapped with the red fluorescence, indicating that the SlRCM1 protein is localized in the chloroplast (Fig. 6d).

### The expression level of *SlRCM1* is directly regulated by the transcription factor SlARF2A

**Fig. 7:**
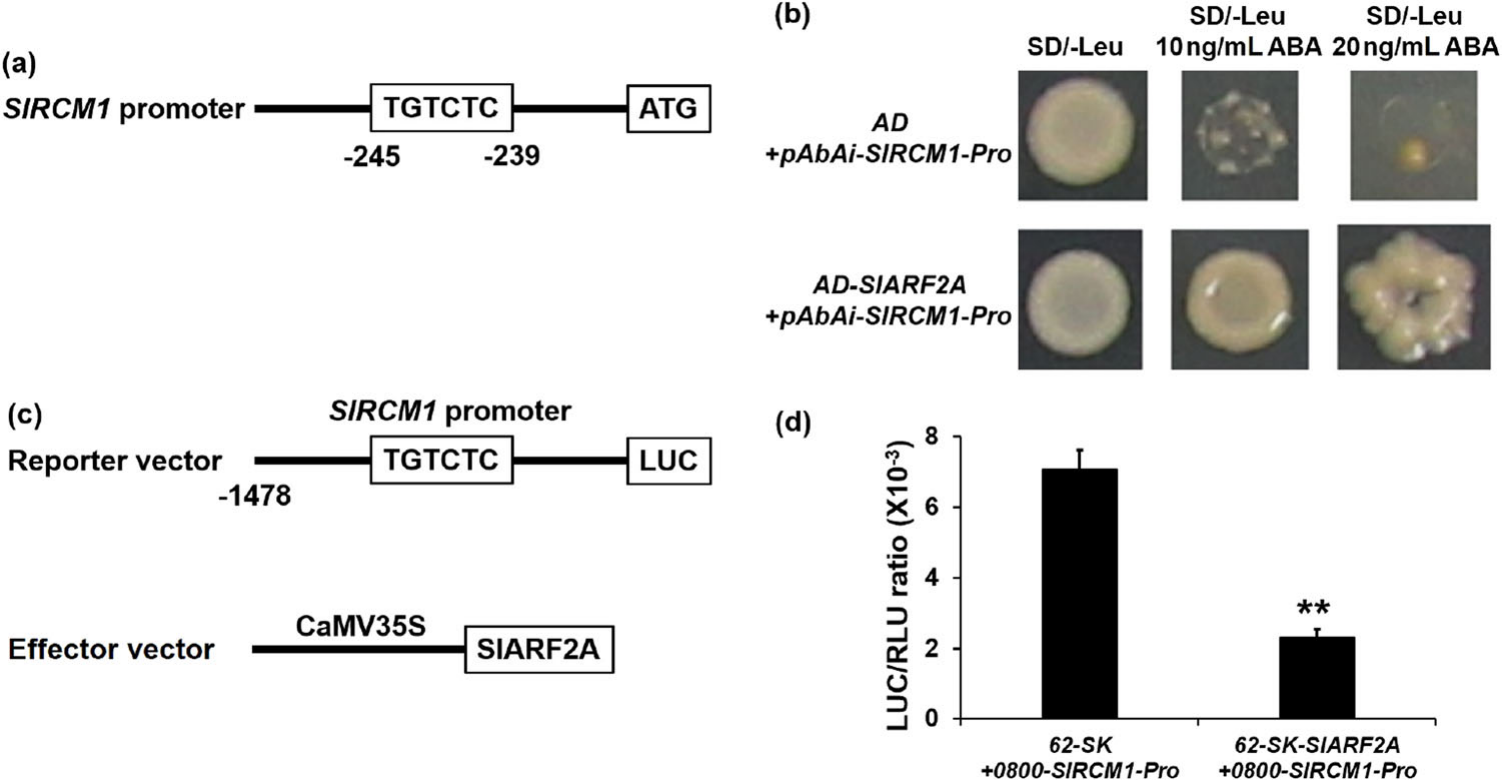
The SlARF2A protein binds to the promoter of *SlRCM1* and downregulates its expression. **a** Schematic representation of the *SlRCM1* promoter and its cis-elements. The sequence in the black box refers to the TGTCTC cis-element. **b** Yeast one-hybrid experiment of SlARF2A protein binding to the promoter of *SlRCM1*. The complete open reading frame (ORF) of *SlARF2A* was cloned into *pGADT7* (*AD*) to generate a *pGADT7-SlARF2A* effector construct. The reporter vector was introduced into yeast strain Y1Hgold together with the effector vector. A combination of the reporter vector and the empty vector *pGADT7* was used as a negative control. The yeast cells were cultured on SD-Leu media supplemented with various concentrations of ABA (0, 10, and 20 ng/ml). **c** Schematic representation of constructs used for the dual luciferase assay. The *SlARF2A* ORF was cloned into *pGreenII 62-SK* to generate a *pGreenII 62-SK-SlARF2A* effector construct. The *SlRCM1* promoter was fused to *pGreenII 0800-LUC* to create a *pGreen II 0800-SlRCM1-Pro* reporter construct. **d** Relative LUC/RLU ratio. The vectors of both the effectors and reporters were transformed into *Agrobacterium* cells, which were then used to infiltrate tobacco leaves. LUC, firefly luciferase activity; RLU, Renilla luciferase activity. The data are presented as the means ± SDs (*n* = 6). The asterisks indicate statistically significant differences according to *t*-tests: **, *P* < 0.01

It was previously reported that ARF proteins regulate gene expression by binding to the TGTCTC cis-element of the target gene promoter^34^. Yeast one-hybrid (Y1H) and dual luciferase experiments indicated that SlARF2A binds to cis-elements of the *SlRCM1* promoter. The TGTCTC cis-element is located -245 to -239 bp upstream of the start codon of *SlRCM1* (Fig. 7a). Yeast cells that were cotransformed with *pGADT7-SlARF2A* and *pAbAi-SlRCM1-Pro* were able to grow on 20 ng/mL aureobasidin A (ABA) SD/-Leu media, but the negative control cotransformed with *pGADT7* and *pAbAi-SlRCM1-Pro* did not grow (Fig. 7b), indicating that the ARF2A protein could directly bind to the promoter of *SlRCM1*.

To further indicate that the SlARF2A protein interacted with the promoter of *SlRCM1* in plants, we constructed a luciferase (LUC) reporter harboring a promoter sequence from -1,478 bp to the start codon (ATG) derived from *SlRCM1* (Fig. 7c). Tobacco leaves were coinfiltrated with *Agrobacterium tumefaciens* (GV3101) strains containing the indicated effector constructs containing SlARF2A and the reporter constructs containing the *SlRCM1* promoter (Fig. 7d). Taken together, these results indicate that SlARF2A physically binds to the *SlRCM1* promoter to downregulate its expression.

## Discussion

Chlorophyll is essential for plants to absorb, transfer and convert light energy to bioenergy and plays a vital role in photosynthesis to promote plant growth and development^35^. Here, we cloned the *SlRCM1* gene, which controls chlorophyll synthesis and chloroplast development in tomato fruits. A single-nucleotide replacement resulting in a premature termination mutation in SlRCM1 impaired chloroplast development in the *rcm1* mutant. Increasing the chlorophyll content in tomato fruits contributes to improved nutrition^36^. Consistently, the soluble solids content of red ripe fruits of *SlRCM1*-overexpressing (OE) lines was significantly higher than that of the wild type, whereas the knockout lines (CR) showed the opposite effect (Fig. S7). In addition, the a*/b* colorimetric value of the ripe red fruits of the *SlRCM1*-overexpressing lines (OE) was significantly higher than that of the wild type, while the knockout lines (CR) showed the opposite effect (Fig. S8). Therefore, characterization of the *SlRCM1* gene may provide insights into chlorophyll synthesis and chloroplast development in tomato fruits.

The *rcm1* mutant was obtained from an EMS-mutagenized population. EMS mutagenesis is widely used in the construction of mutant libraries and in plant functional genomics^37,38^. For instance, the *hst1* mutant generated from EMS mutagenesis was crossed with the wild type to map the *OsRR22* gene^39^. TILLING analysis^40^ of an EMS-mutagenized population of *Arabidopsis* showed that EMS mutagenesis produces a large number of single-base substitutions within hotspot segments^41^. In this study, a reduced-chlorophyll mutant, *rcm1*, was acquired by EMS mutagenesis and crossed with wild type to map the *SlRCM1* gene responsible for the yellowish-fruit–producing mutant. Due to the close genetic background between the EMS-induced mutant and its wild type, crossing a mutant with its wild type is a fast and effective approach to isolate a gene^42,43^. Two SNPs in the recessive pool with an allele frequency of 1 were found between SL2.50ch08_1 and SL2.50ch08_1010000 (Table S2). Since the SNP at SL2.50ch08_ 394612 was located in an intergenic region, we speculated that it is not a causal SNP for the reduced chlorophyll phenotype of *rcm1*. The SNP (A → T) in the second exon of *Solyc08g005010* resulted in a premature stop codon (Fig. 2e). Furthermore, the *rcm1* mutant phenotype was rescued by overexpression of the *SlRCM1* gene in the *rcm1* mutant, which confirmed that *SlRCM1* was responsible for the mutant phenotype (Fig. 3). MutMap analysis could therefore be a feasible approach to genetically identify genes in EMS-mutagenized mutants.

SlRCM1 has highly conserved functions in both chloroplast development and chlorophyll synthesis. There are two homologous genes of *SlRCM1* in *Arabidopsis*: *BCM1* and *BCM2*^22^. SlRCM1 was found to share 78% amino acid sequence identity with BCM1 and 84% identity with BCM2 (Fig. S1). Previous studies have shown that *BCM1* and *BCM2* regulate chlorophyll synthesis and chloroplast development in *Arabidopsis*^22^. Yeast two-hybrid (Y2H) assays, bimolecular fluorescence complementation (BiFC) assays, coimmunoprecipitation (Co-IP) assays and enzyme activity-measuring experiments in *Arabidopsis* demonstrated that BCM1 interacts with GUN4 to stimulate Mg-chelatase activity and optimize chlorophyll synthesis^22^. BCM1 also interacts with SGR to prevent chlorophyll degradation^22^. *SlRCM1*’s ortholog in soybean is the stay-green *G* gene and regulates chlorophyll synthesis and chloroplast development in the seed coat of soybean^25^. However, biological divergence has shown that *SlRCM1* regulates chlorophyll synthesis and chloroplast development in fruits, while its ortholog regulates chlorophyll synthesis in the seed coat of soybean^25^ and in the leaves of *Arabidopsis*^22^.

It has been reported that *SlRCM1* orthologs modulate chlorophyll synthesis in the leaves of *Arabidopsis* and in the seed coat of soybean^22,25^. *BCM1* overexpression did not alter the chlorophyll content in *Arabidopsis thaliana*, but chlorophyll accumulation was inhibited only in the leaves of the *bcm1* mutant^22^. In the present study, fruits of the tomato *SlRCM1* knockout lines showed a yellowish phenotype due to impaired chloroplasts. The chlorophyll content in the MG-stage fruits of the *SlRCM1*-overexpressing lines increased significantly. Moreover, chlorophyll was also detected in the RR-stage fruits of the *SlRCM1* overexpression lines (Fig. 4). Taken together, these results showed that *SlRCM1* has a diversified and strong effect on chlorophyll synthesis and chloroplast development in fruits.

In summary, we have demonstrated that *SlRCM1*, a chloroplast-targeted ortholog of the BCM1 protein in *Arabidopsis* and stay-green G protein of soybean, participates in chlorophyll synthesis and chloroplast development in tomato fruits. *SlRCM1* modulates the number of thylakoids and the structure of thylakoid membranes in chloroplasts. *SlRCM1* was identified as the causal gene at the *Lutescent1* locus. An understanding of *SlRCM1* provides insights into the molecular mechanism underlying fruit development and target genes for genetic improvement in horticultural crop species.

## Materials and methods

### Plant materials and mutant screening

A chlorophyll-deficient mutant (*rcm1*) was derived from EMS mutagenesis of the processed tomato inbred line Ligeer 87-5. For EMS mutagenesis, Ligeer 87-5 seeds were immersed in 1% EMS solution and shaken in a shaker for 12 h. The seeds were then rinsed with running water for 10 min and germinated in an artificial incubator at 30 °C. Mutants were screened in the M_2_ generation^44^. We constructed an F_2_ mapping population by crossing Ligeer 87-5 with *rcm1*. The *l1* mutant (LA3717) in the AC background was obtained from the TGRC (https://tgrc.ucdavis.edu). For the overexpression constructs, *SlRCM1* was amplified and cloned into a pHELLSGATE8 vector driven by the *CaMV35S* promoter and its native promoter^45^. The overexpression vector was introduced into *Solanum lycopersicum* cv. AC and the *rcm1* and *l1* mutants through *Agrobacterium*-mediated transformation. *SlRCM1* knockout mutants in the AC background were generated using the CRISPR/Cas9 system^46^. The 2934-bp promoter of the *SlRCM1* gene was amplified from AC and cloned into a pV3P vector driving *β*-glucosidase (GUS), yielding a Pro^*SlRCM1*^::GUS vector, which was subsequently transformed into AC. The tomato tissues were quickly frozen in liquid nitrogen and stored at −80 °C. The tomato plants used in this study were grown in a greenhouse, and the primers used in this experiment are listed in Table S4.

### Transmission electron microscopy

The fruits of Ligeer 87-5, the *rcm1* mutant, AC and the transgenic lines were isolated and fixed in 0.05 M cacodylate buffer consisting of 2% glutaraldehyde and dehydrated in ethanol. After embedding in Spurr resin, ultrathin sections of the samples were obtained using a Leica EMUC6 ultramicrotome. A Hitachi H-7650 transmission electron microscope was used to observe the ultrastructure of the plastids.

### Determination of the chlorophyll content

For chlorophyll extraction, fruits of Ligeer 87-5, the *rcm1* mutant, AC and the transgenic lines at different developmental stages were placed in 10 mL of 80% (v/v) acetone in the dark until the tissues became white. Their absorbance was subsequently measured at 646, 663 and 470 nm, and the chlorophyll content was measured and calculated as previously reported^47^.

### BSA and DNA-seq

An F_2_ population comprising 307 individuals was derived from a cross between Ligeer 87-5 and the *rcm1* mutant. In this population, equal amounts of DNA were pooled from 25 plants with a wild-type phenotype and 25 plants with a mutant phenotype. Approximately 25× genome sequences for each pool were generated using the Illumina HiSeq X Ten platform^48^. Due to the close genetic background of the M82 and Ligeer 87-5 processed tomato genotypes, the M82 genome version SL 2.50 (http://solgenomics.net) was used as the reference genome to facilitate mining of the causal SNPs.

### Subcellular localization

The *SlRCM1* coding sequence without the stop codon was amplified from the cDNA of AC and then cloned into a *pHBT* vector driven by the *CaMV35S* promoter, yielding CaMV35S::SlRCM1-GFP. This vector was subsequently transformed into *Agrobacterium tumefaciens* strain GV3101, which was then injected into the leaves of *Nicotiana benthamiana* (*N. benthamiana*) as previously described^49^. After 48 h of incubation at 25 °C, the fluorescence of GFP and RFP in the tobacco leaves was observed using Leica Confocal software. Chloroplasts that exhibit red fluorescence were used as positive controls. The primers used in this experiment are listed in Table S4.

### Quantitative reverse transcription PCR

Total RNA was extracted from frozen tissues using TRIzol reagent (Vazyme, Nanjing, China). RNA was then reverse transcribed into cDNA using a first-strand cDNA Synthesis Kit (Vazyme). The product length ranged from 80 to 200 bp. The qRT-PCR was used to determine the transcript levels of genes in 96-well plates with a Roche LightCycler^®^ 480 system according to the manufacturer’s protocol^32^. The expression of the *ACTIN* gene (*Solyc11g005330*) was used as an internal control. The primers used were designed using Primer Premier 5 (Table S4).

### Colorimeter-based evaluation of red ripe fruits

The a* and b* values of ripe red fruits were measured using a CM-5 colorimeter. Three independent points were determined for each fruit assay, and six fruits were measured per line.

### Measurement of total soluble solids

The total soluble solids (Brix) of red ripe fruits were measured using a digital refractometer (PR100, Atago Co., Ltd.). All sample assays were performed for three technical replicates and six biological replicates.

### Ethylene assays

Tomato fruits were collected at 37, 43 and 49 days after flowering. The determination of ethylene content in the tomato fruits was based on previous methods^50^. All the samples consisted of three technical replicates and three biological replicates.

### Pollen viability assays

The pollen of blooming flowers was soaked in I-KI solution (1% KI and 0.5% I_2_) for 2 min. The stained pollen was then examined under a low-magnification microscope. The viable pollen was stained dark blue by I-KI.

### Chlorophyll fluorescence measurements

After the leaves of Ligeer 87-5, the *rcm1* mutant, AC and *L1*-CR were conditioned in the dark for 30 min, an imaging pulse amplitude modulated chlorophyll fluorimeter (IMAG-MAXI; Heinz Walz, Effeltrich, Germany) was used to determine chlorophyll fluorescence. The maximal photochemical efficiency of PSII (Fv/Fm) and the quantum efficiency of PSII (Y(II)) were calculated and determined according to previously reported methods^51^.

### Yeast one-hybrid assays

The *SlARF2A* ORF was cloned into a *pGADT7* vector to yield a prey construct. The *SlRCM1* promoter was inserted into a *pAbAi* vector to yield a bait construct. The *pAbAi* bait vector was then used to transform yeast strain Y1HGold, which was integrated into the yeast genome to generate reporter strains. The prey vector was introduced into the reporter strains and grown for three days on SD/-Leu-Ura media. The positive yeast strains were selected and diluted in double-distilled water to an OD600 of 0.1, and 2 μL of the suspension was spotted onto SD/–Leu media with or without ABA (0, 10 and 20 ng/mL), followed by 3 days of incubation at 30 °C. *pGADT7* and *pAbAi-PSY1-Pro* served as negative controls. The primers used in this experiment are listed in Table S4.

### Dual luciferase transactivation assays

To generate an effector construct, the full-length *ARF2A* ORF was inserted into a *pGreenII 62-SK* vector. Similarly, the promoter from *SlRCM1* was inserted into *pGreenII 0800-LUC* to yield a reporter construct. The constructs and the pSoup helper plasmid were simultaneously introduced into *Agrobacterium tumefaciens* (GV3101). Tobacco leaves were infiltrated with the *Agrobacterium* strains and harvested three days later. The firefly LUC and Renilla luciferase (RLU) activities were quantified using a dual-luciferase reporter assay system. The transactivation activities were expressed as the ratio of LUC to RLU activity. The primers used in this experiment are listed in Table S4.

## Supplementary information

Supplymentary Tables S1-S4

Supplymentary Figures S1-S8
