# Supplementary material for: SlRCM1, which encodes tomato Lutescent1, is required for chlorophyll synthesis and chloroplast development in fruits
Source: Hortic Res. 2021 Jun 1;8:128. doi: 10.1038/s41438-021-00563-6 (PMC8166902; doi:10.1038/s41438-021-00563-6)
Supplement: Supplementary file 2 — Supplymentary Figures S1-S8 [file 41438_2021_563_MOESM2_ESM.pdf]

Supplementary figures

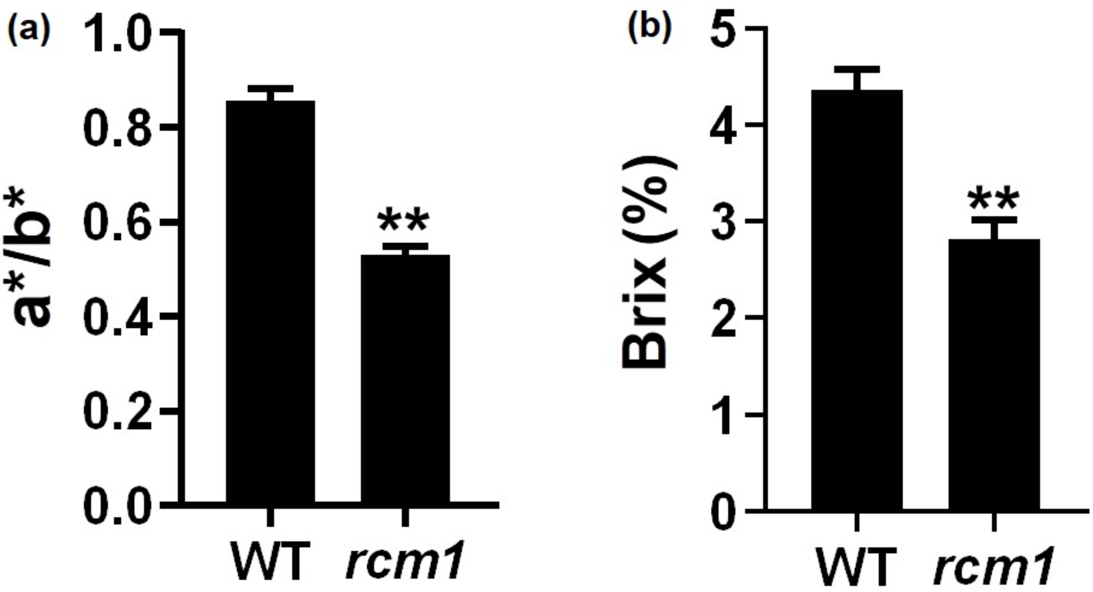

Fig. S1 Quality characteristics of red ripe fruits of the *rcm1* mutant.

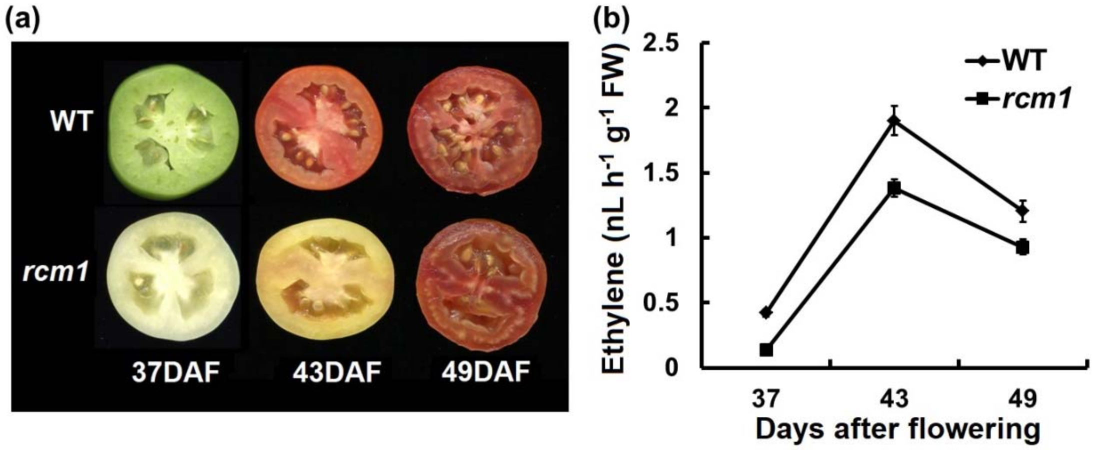

Fig. S2 Delayed fruit ripening of the *rcm1* mutant.

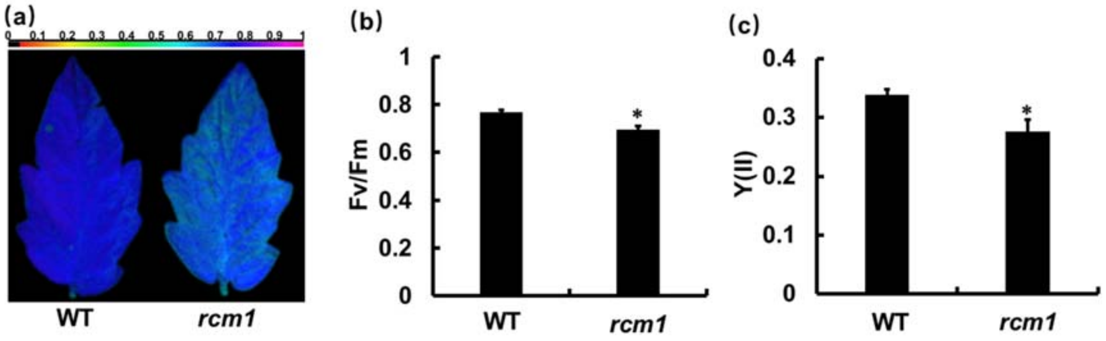

Fig. S3 Photosynthesis capacity of wild-type (Ligeer 87-5) and *rcm1* mutant leaves.

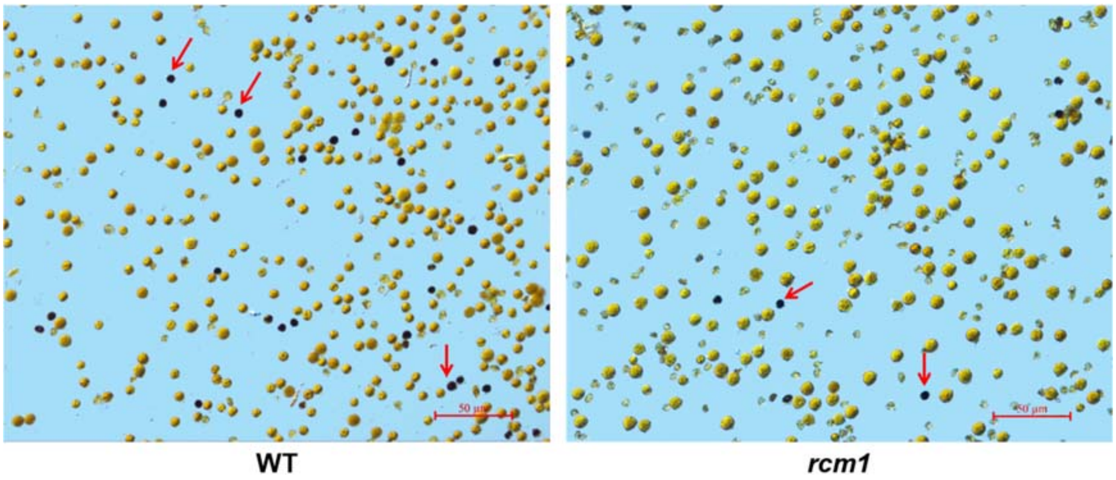

Fig. S4 Viability of pollen from the WT (Ligeer 87-5) and *rcm1* mutant.

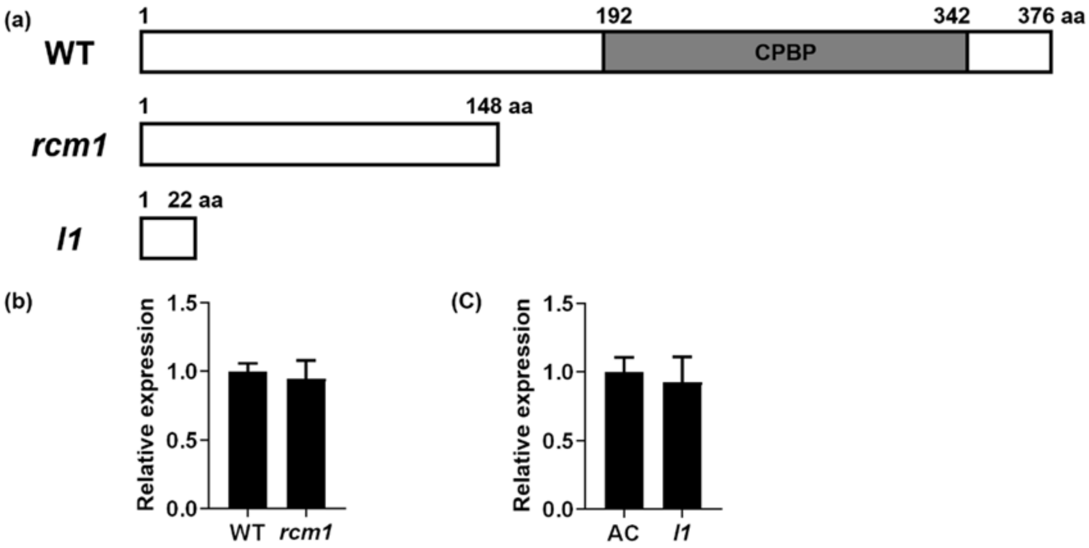

Fig. S5 Conserved domain of SlRCM1 and the expression level of the N-terminal protein.

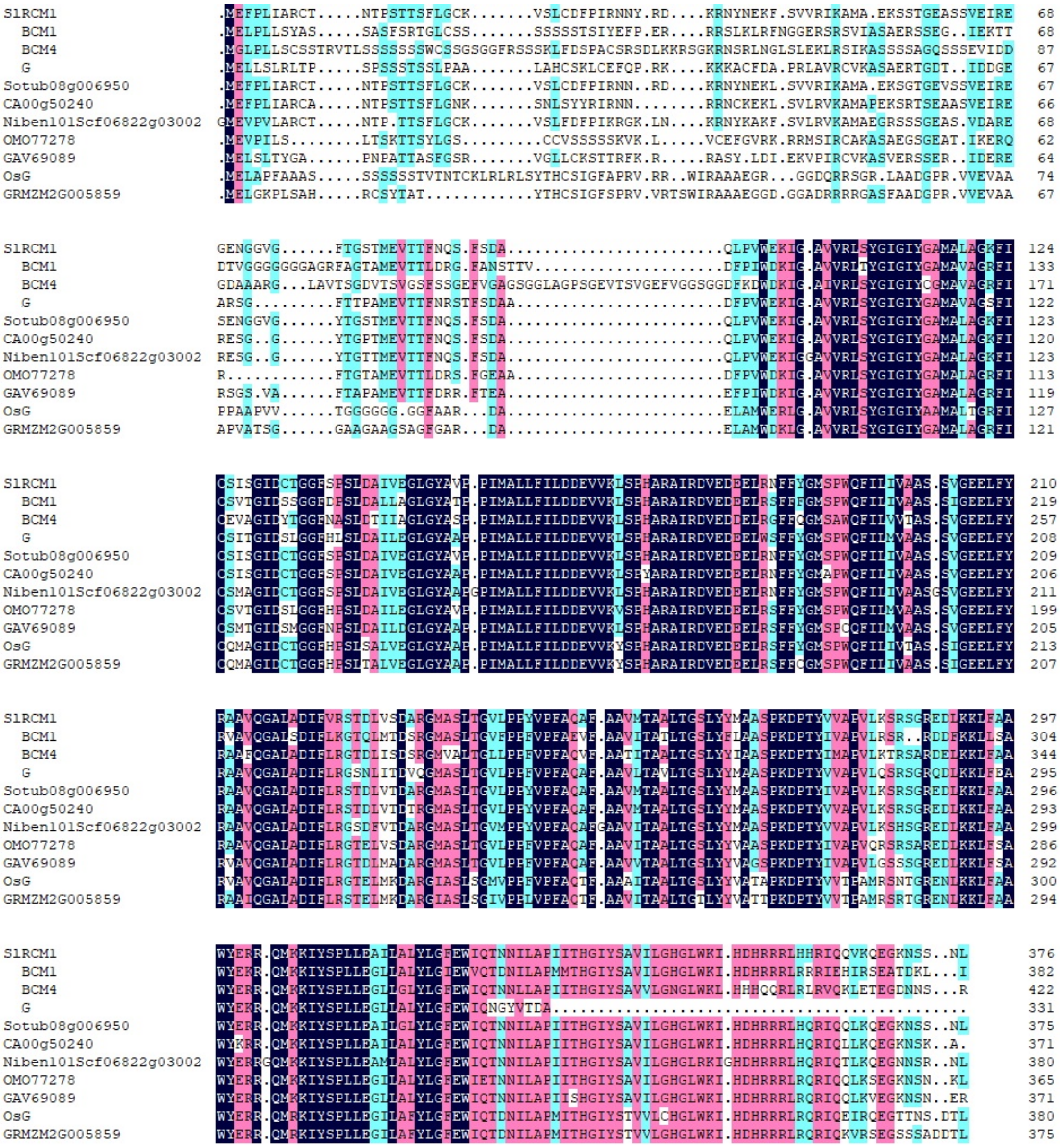

Fig. S6 Amino acid sequence alignment of chloroplast-targeted metalloendopeptidases from different species.

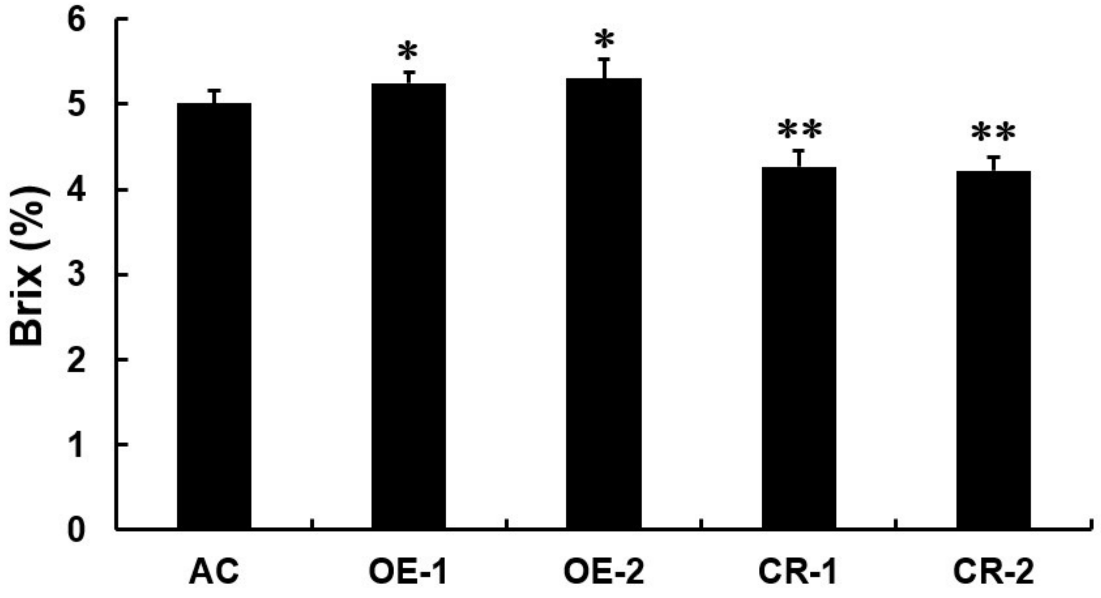

Fig. S7 Accumulation of total soluble solids in RR-stage fruits of wild-type (AC) and transgenic lines.

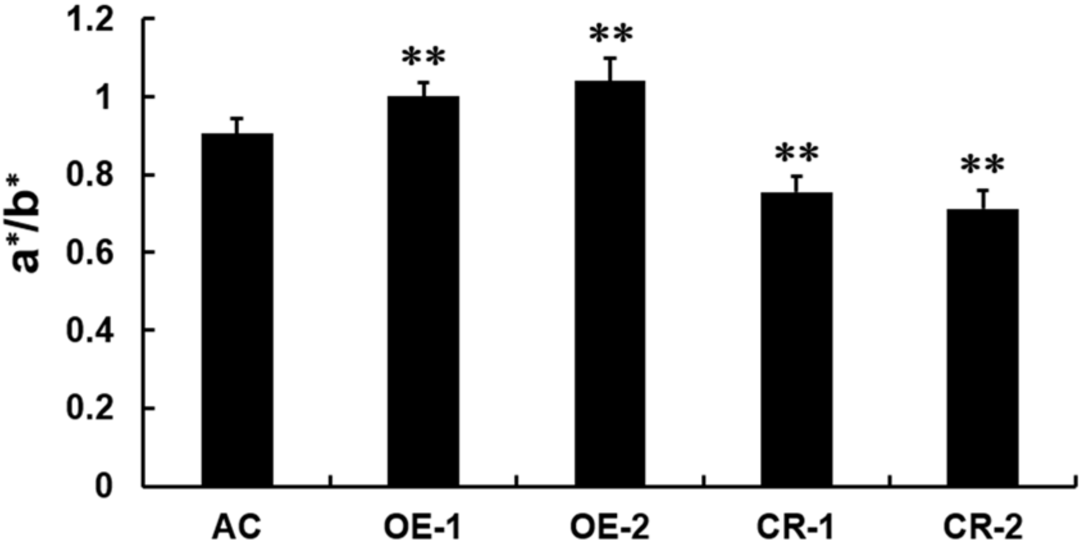

Fig. S8 Colorimetric parameters (a* and b*) of fruits of wild-type (AC) and transgenic lines as determined by a colorimeter (cm-5) at the RR stage.
